# GERI-MOBILE HEALTH: DEVELOPMENT AND FEASIBILITY OF A PROGRAM TO HELP OLDER VETERANS USE VA MENTAL HEALTH MOBILE APPS

**DOI:** 10.1093/geroni/igz038.2072

**Published:** 2019-11-08

**Authors:** Christine E Gould, Flora Ma, Ashley N Scales, Christine Juang, Chalise Carlson, Julia Loup, Erin Sakai

**Affiliations:** 1 VA Palo Alto Health Care System, Palo Alto, United States; 2 VA Palo Alto, Palo Alto, California, United States; 3 University of Alabama, Tuscaloosa, Alabama, United States

## Abstract

VA mental health self-management mobile applications (apps) teach evidence-based skills such as mindfulness and behavior activation. Older Veterans are likely to benefit from learning these skills, but many need assistance learning to use their mobile devices. To bridge this knowledge gap, we developed a clinical program (Geri-Mobile Health) and accompanying patient education materials to help older Veterans use apps to meet their mental health and well-being goals. The program provides personalized coaching support consisting of (1) teaching basic mobile device use; (2) selecting a goal-consistent app; and (3) encouraging behavior change using the app. The presentation will describe the preliminary outcomes and implementation process. Initial results suggest that the program is feasible, acceptable, and may increase mobile device proficiency for novice users. Additionally, we disseminated 1,418 sets of education materials to providers in 21 states. Challenges and successes at the local and national levels will be discussed.

